# Identification of the Nerve-Cancer Cross-Talk-Related Prognostic Gene Model in Head and Neck Squamous Cell Carcinoma

**DOI:** 10.3389/fonc.2021.788671

**Published:** 2021-11-29

**Authors:** Jun Li, Yunhong Xu, Gang Peng, Kuikui Zhu, Zilong Wu, Liangliang Shi, Gang Wu

**Affiliations:** Cancer Center, Union Hospital, Tongji Medical College, Huazhong University of Science and Technology, Wuhan, China

**Keywords:** nerve, prognostic model, HNSC, neurotransmitter, bioinformatics

## Abstract

The incidence of head and neck squamous cell carcinoma (HNSC) is increasing year by year. The nerve is an important component of the tumor microenvironment, which has a wide range of cross-talk with tumor cells and immune cells, especially in highly innervated organs, such as head and neck cancer and pancreatic cancer. However, the role of cancer-nerve cross-talk-related genes (NCCGs) in HNSC is unclear. In our study, we constructed a prognostic model based on genes with prognostic value in NCCGs. We used Pearson’s correlation to analyze the relationship between NCCGs and immune infiltration, microsatellite instability, tumor mutation burden, drug sensitivity, and clinical stage. We used single-cell sequencing data to analyze the expression of genes associated with stage in different cells and explored the possible pathways affected by these genes *via* gene set enrichment analysis. In the TCGA-HNSC cohort, a total of 23 genes were up- or downregulated compared with normal tissues. GO and KEGG pathway analysis suggested that NCCGs are mainly concentrated in membrane potential regulation, chemical synapse, axon formation, and neuroreceptor-ligand interaction. Ten genes were identified as prognosis genes by Kaplan-Meier plotter and used as candidate genes for LASSO regression. We constructed a seven-gene prognostic model (NTRK1, L1CAM, GRIN3A, CHRNA5, CHRNA6, CHRNB4, CHRND). The model could effectively predict the 1-, 3-, and 5-year survival rates in the TCGA-HNSC cohort, and the effectiveness of the model was verified by external test data. The genes included in the model were significantly correlated with immune infiltration, microsatellite instability, tumor mutation burden, drug sensitivity, and clinical stage. Single-cell sequencing data of HNSC showed that CHRNB4 was mainly expressed in tumor cells, and multiple metabolic pathways were enriched in high CHRNB4 expression tumor cells. In summary, we used comprehensive bioinformatics analysis to construct a prognostic gene model and revealed the potential of NCCGs as therapeutic targets and prognostic biomarkers in HNSC.

## Introduction

Head and neck squamous cell carcinoma (HNSC) is the sixth most common cancer in the world, with 890,000 new cases and 450,000 deaths in 2018 (1). Frighteningly, the incidence of HNSC continues to rise and is forecasted to increase by 30% in 10 years (2). Surgery, radiotherapy, chemotherapy, and immunotherapy are the main methods for the treatment of HNSC (3). The 5-year survival rate of HNSC has improved, reaching 66% at the beginning of the twenty-first century (4).

Previous studies have shown that PIK3CA, TP53, CDKN2A, and other genes play an important role in the occurrence and development of HNSC (5). However, the research on prognostic gene signatures of HNSC is far from enough, and it is of great significance to explore the molecular mechanism of HNSC.

Nerve is an important part of the tumor microenvironment. Recent studies have shown that the interaction of peripheral nerves (sympathetic, parasympathetic, and sensory nerves) with tumor cells and interstitial cells promotes the occurrence and development of various solid tumors and hematological malignancies (6). Tumor prognosis is related to nerve infiltration, which is most common in highly innervated organs (7). In HNSC, nerve invasion is an independent prognostic factor (8), with an incidence of 25%–80% (9). In addition, tumor may reactivate nerve development and regeneration to promote their growth and survival (10). The prognostic value of cancer-nerve cross-talk-related genes (NCCGs) in HNSC has not been studied.

In this study, bioinformatics analysis was used to study the expression and prognostic value of NCCGs and related regulatory axes. Our data may provide evidence for new biomarkers and therapeutic targets.

## Materials and Methods

### Data and Processing

RNA sequencing data and clinical information of 501 HNSC patients were derived from the TCGA database and downloaded from UCSC (https://xenabrowser.net/datapages/). RNA sequencing data and survival information of 97 HNSC patients in GSE41613 were derived from the Gene Expression Omnibus (GEO) database (https://www.ncbi.nlm.nih.gov/geo/query/acc.cgi?acc=GSE41613). Clinical phenotypic information and processed analysis data are shown in **Supplementary Tables S1**, **S3**. Single-cell sequencing data of HNSC were derived from the GEO database (https://www.ncbi.nlm.nih.gov/geo/query/acc.cgi?acc=GSE103322). Gene expression profiling of 14 oral lichen planus (OLP) epithelia and 14 normal oral epithelia were derived from the GEO database (https://www.ncbi.nlm.nih.gov/geo/query/acc.cgi?acc=GSE52130, https://www.ncbi.nlm.nih.gov/geo/query/acc.cgi?acc=GSE38616). Use R software (4.0) for data processing and analysis.

### Identification of Nerve-Cancer Cross-Talk-Related Genes

Forty-two nerve-cancer cross-talk genes were identified by previous references (6, 7), and these genes are displayed in **Supplementary Table S2**.

### Mutation Analysis and Protein-Protein Interaction

The cBioPortal for Cancer Genomics (http://cbioportal.org) provides a Web resource for exploring, visualizing, and analyzing multidimensional cancer genomics data (11), by which we implemented the mutation analysis. The STRING database aims to integrate all known and predicted associations between proteins, including both physical interactions as well as functional associations (12). Our protein-protein interaction (PPI) analysis of 42 NCCGs was performed by STRING (https://string-db.org/).

### Functional Enrichment Analysis

Both Gene Ontology (GO) analysis and Kyoto Encyclopedia of Genes and Genomes (KEGG) analysis were plotted by an R package Pathview, which is a novel tool set for pathway-based data integration and visualization (13).

### Construction of Prognostic Gene Model

The least absolute shrinkage and selection operator (LASSO) regression algorithm was used for feature selection, and 10-fold cross-validation was used. Log-rank was used to test K-M survival analysis to compare the survival differences between the two groups, and time-ROC analysis was performed to evaluate the accuracy of the prediction. The above analysis was done by the R package glmnet (14). For the nomogram model, univariate and multivariate regression analyses were used for finding genes and clinical phenotype with prognostic value, and the forest map was completed by the R package forestplot. The R package rms was used for establishing a nomogram to predict the 1-, 3-, and 5-year survival rates.

### Immune Infiltration, Tumor Mutation Burden, Microsatellite Instability, and Drug Sensitivity

TIMER (https://cistrome.shinyapps.io/timer/) is a web server for comprehensive analysis of tumor-infiltrating immune cells (15), by which we explored the relationship between gene expression and immune infiltration. We used Spearman’s correlation analysis to describe the relationship between gene expression and tumor mutation burden (TMB), microsatellite instability (MSI) in HNSC *via* the R package ggstatsplot.

The Cancer Therapeutics Response Portal (CTRP) links genetic, lineage, and other cellular features of cancer cell lines (CCL) to small-molecule sensitivity to accelerate the discovery of patient-matched cancer therapeutics, including the relationship of 481 compounds and 860 CCLs (16). GSCALite is a user-friendly web server for dynamic analysis and visualization of gene set in cancer and drug sensitivity correlation (17). We used the CTRP drug analysis module of GSCALite (http://bioinfo.life.hust.edu.cn/GSCA/#/drug) to analyze the relationship between NCCG gene expression and drug sensitivity in pan-cancer (17).

### Gene Expression in Different Cells of HNSC

Tumor immune single-cell hub (TISCH, http://tisch.comp-genomics.org/) is a single-cell RNA-seq database focusing on tumor microenvironment (18). We used this webtool to explore gene expression in different cells of HNSC.

### Identification the Function of Genes by Gene Set Enrichment Analysis (GSEA)

Gene set enrichment analysis (GSEA) (available at http://software.broadinstitute.org/gsea/index.jsp) is a method to interpret gene expression data by focusing on gene sets (19). This method was used for identifying enriched KEGG pathways in cells with high gene expression, compared with cells with low gene expression.

## Results

### Expression and Mutation of NCCGs in HNSC

**Figure 1:**
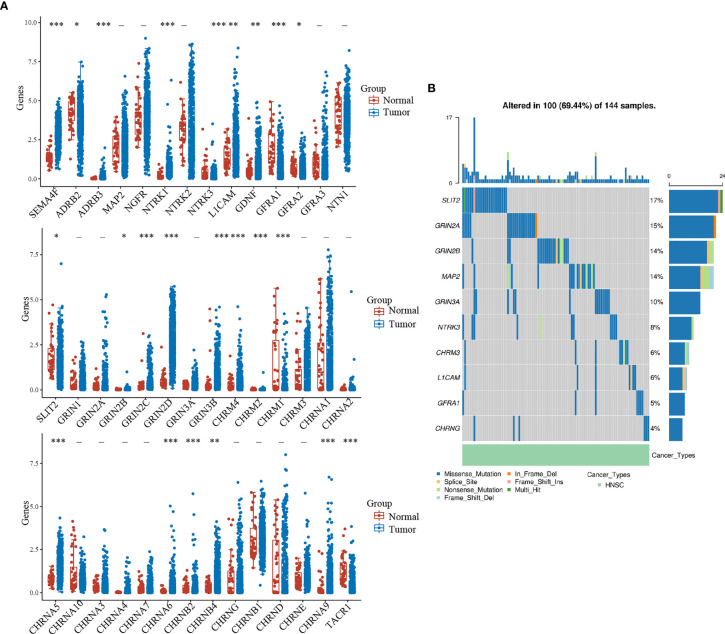
Expression and mutation landscape of NCCGs. **(A)** The expression of 42 NCCGs in HNSC and normal tissue. **(B)** The mutation landscape of the top 10 mutation rate of NCCGs. ^*^ *p* < 0.05, ^**^ *p* < 0.01, ^***^ *p* < 0.001. NCCGs, nerve-cancer cross-talk genes; HNSC, head and neck squamous cell carcinoma.

First, we explored the expression of 42 NCCGs in HNSC and normal tissue in the TCGA database. A total of 23 genes were up- or downregulated in HNSC (**Figure 1A**). Compared with normal tissue, SEMA4F, ADRB2, ADRB3, NTRK1, NTRK3, LICAM, GDNF, GFRA2, GRIN2B, GRIN2C, GRIN2D, GRIN3B, CHRM2, CHRM4, CHRNA5, CHRNA6, CHRNA9, CHRNB2, and CHRNB4 were upregulated, while GFRA1, SLIT2, CHRM1, TACR1 were downregulated (^*^ *p* < 0.05, ^**^ *p* < 0.01, ^***^ *p* < 0.001).

We then summarized the incidence of copy body mutation and somatic mutation of NCCGs in HNSC (**Figure 1B**). In the 144 samples, NCCGs were altered in 100 (69.44%) samples. The most common type of mutation is missense mutation (blue band). We showed the top 10 genes with the higher mutation rate; SLIT2 (17%) had the highest mutation rate, followed by GRIN2A, GRIN2B, MAP2, GRIN3A, NTRK3, CHRM3, L1CAM, GFRA1, and CHRNG.

### Functional Enrichment of NCCGs

**Figure 2:**
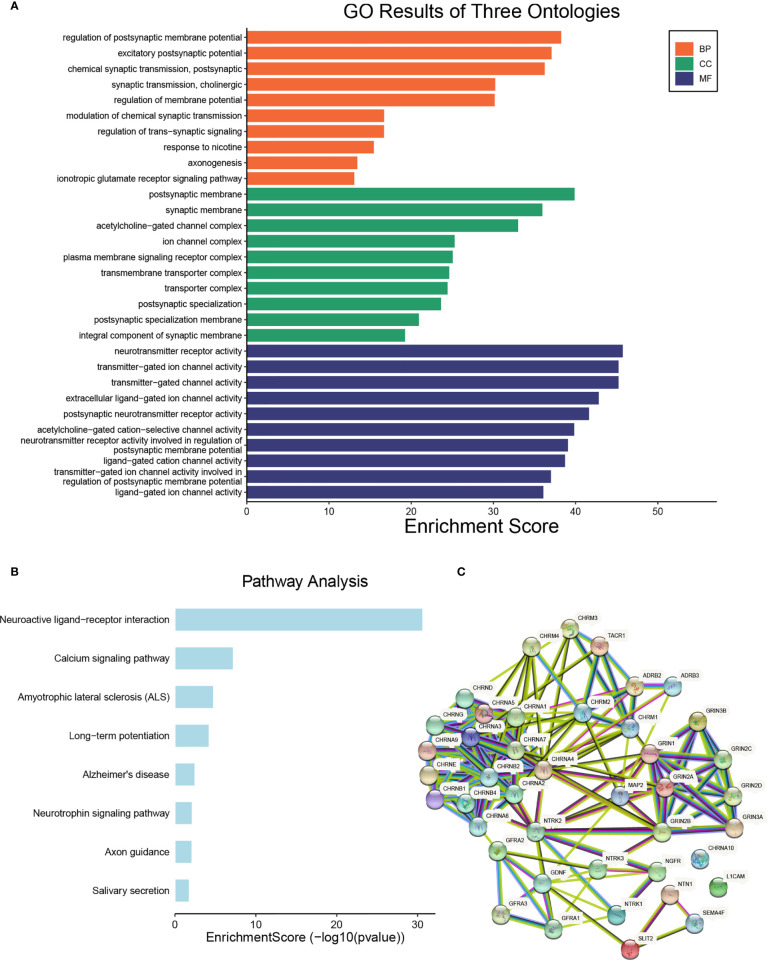
The functional enrichment analysis of NCCGs. **(A)** The Gene Ontology (GO) analysis. **(B)** The Kyoto Encyclopedia of Genes and Genomes (KEGG) analysis. **(C)** The protein-protein interaction (PPI) of NCCGs. NCCGs, nerve-cancer cross-talk genes.

To further explore the function of NCCGs, we used GO analysis and KEGG pathway analysis. Through GO analysis, we found that 42 NCCGs were mainly enriched in membrane potential regulation, chemical synapse, axon formation, and so on (**Figure 2A**). In addition, KEGG pathway analysis showed that 42 NCCGs were mainly involved in neuroreceptor-ligand interaction, calcium signal pathway, and so on (**Figure 2B**). Finally, we did the PPI analysis of 42 NCCGs, and the results showed a complex interaction between these genes (**Figure 2C**).

### Construction and Verification of Prognostic Gene Model

**Figure 3:**
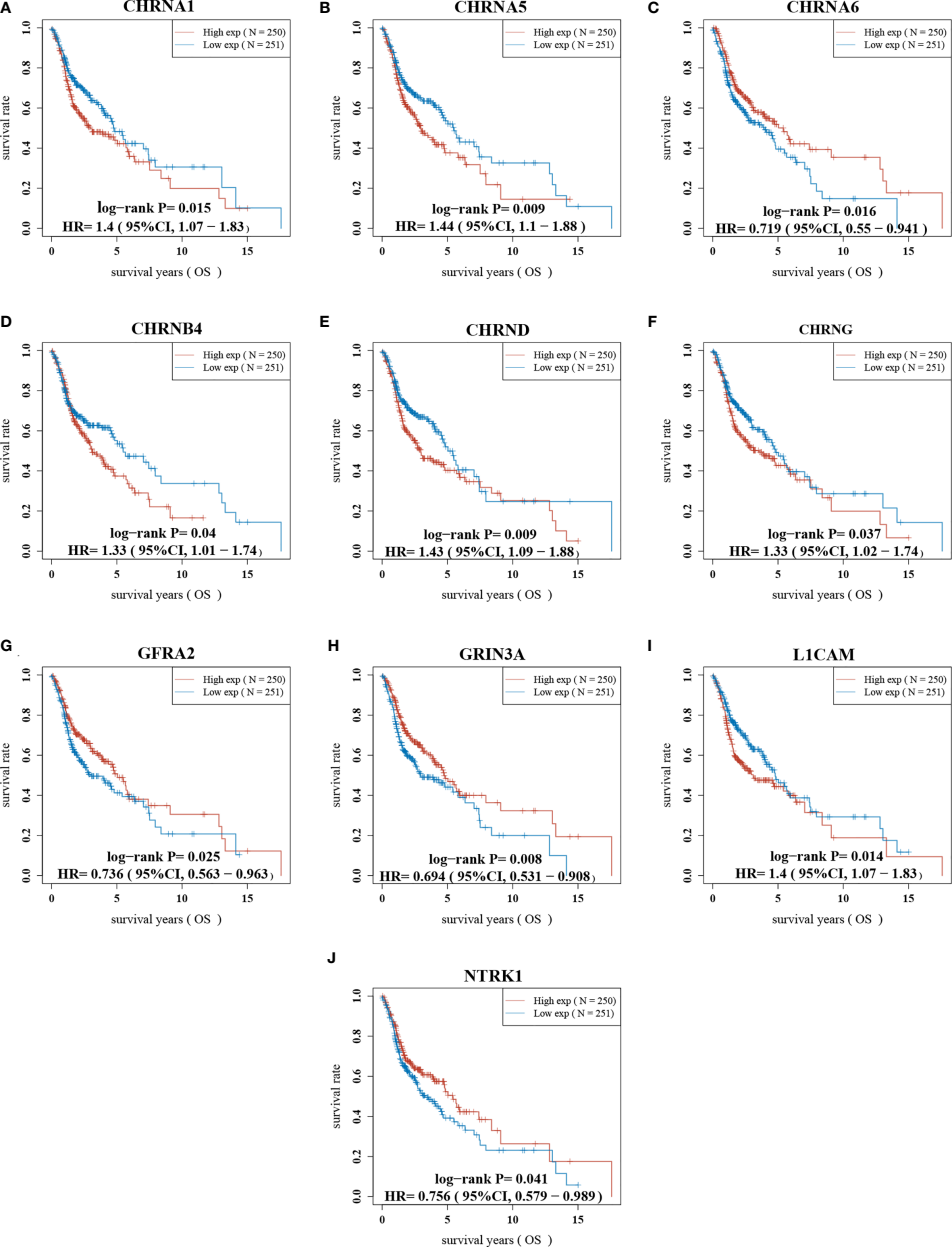
The prognostic value of NCCGs in HNSC. The overall survival of CHRNA1 **(A)**, CHRNA5 **(B)**, CHRNA6 **(C)**, CHRNB4 **(D)**, CHRND **(E)**, CHRNG **(F)**, GFRA2 **(G)**, GRIN3A **(H)**, L1CAM **(I)**, and NTRK1 **(J)** in HNSC patients in the high/low-expression groups. NCCGs, nerve-cancer cross-talk genes; HNSC, head and neck squamous cell carcinoma.

First of all, we looked for prognostic genes in 42 NCCGs by K-M plotter. A total of 10 genes with prognostic value are shown in **Figure 3**. The results showed that the high expression of CHRNA1, CHRNA5, CHRNB4, CHRND, CHRNG, and LICAM and the low expression of CHRNA6, GFRA2, GRIN3A, and NTRK1 in HNSC associated with a poor prognosis.

**Figure 4:**
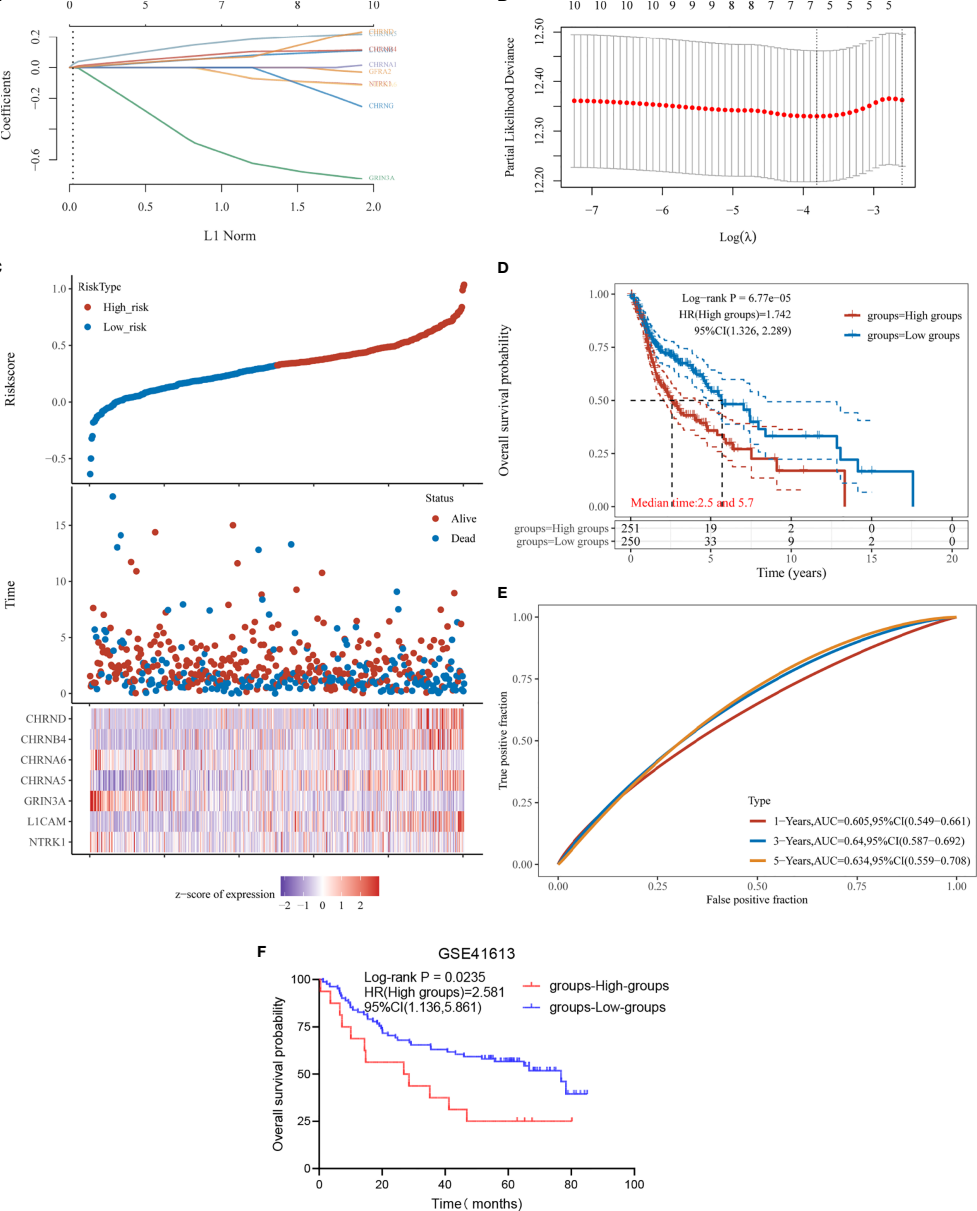
Construction and verification of prognostic gene model. **(A)** LASSO coefficient profiles of the seven NCCGs. **(B)** Plots of the 10-fold cross-validation error rates. **(C)** Distribution of risk score, survival status, and the expression of seven NCCGs in HNSC. **(D, E)** Overall survival curves for HNSC patients in the high/low-risk score group and the ROC curve of measuring the predictive value. **(F)** Overall survival rate for 97 HNSC patients of GSE41613 in the high/low-risk score groups. NCCGs, nerve-cancer cross-talk genes; HNSC, head and neck squamous cell carcinoma.

Next, based on these 10 prognostic genes, we used LASSO regression analysis to establish a prognostic gene model (**Figure 4**). A total of seven genes were included in the model, and risk score = (−0.0117) * NTRK1+ (0.057) * L1CAM+ (−0.5121) * GRIN3A+ (0.1541) * CHRNA5+ (−0.0146) * CHRNA6+ (0.0795) * CHRNB4+ (0.0564) * CHRND. We calculated the risk score for each HNSC patient and divided them into the high-score group and the low-score group (**Supplementary Table S3**). The risk-score distribution, survival status, and the expression of seven genes are shown in **Figure 4C**. The K-M plotter showed that the prognosis of the high-score group was worse than that of the low-score group (**Figure 4D**). The AUCs of the 1-, 3-, and 5-year ROC curve was 0.605, 0.64, and 0.634, respectively (**Figure 4E**).

Finally, we used data of 97 HNSC patients in GSE41613 to verify the effectiveness of the model. According to the risk score, the patients were divided into high-score group and low-score group (**Supplementary Table S4**), and the prognosis was compared by K-M plotter. The results showed that the model could distinguish the prognosis of patients (*p* = 0.0235, **Figure 4F**).

### Establishment of the Prognostic Nomogram

**Figure 5:**
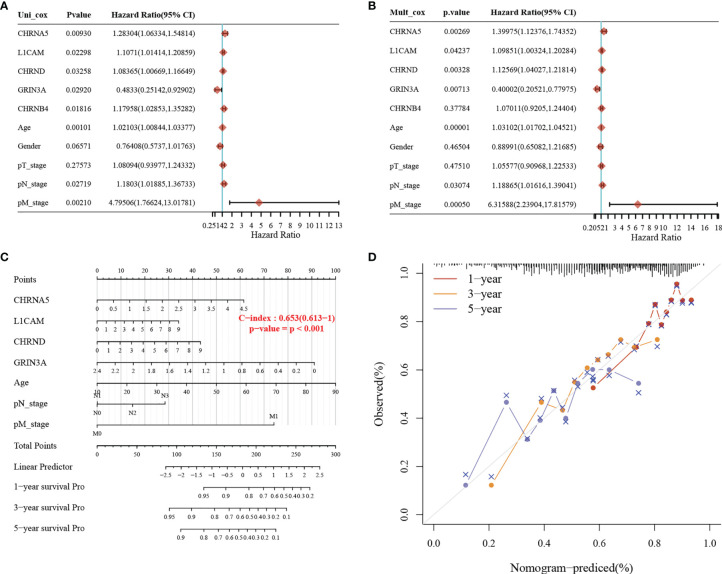
Construction of a predictive nomogram. **(A, B)** Hazard ratio and *p*-value of the constituents involved in univariate and multivariate Cox regression considering clinically the parameters and seven prognostic NCCGs in HNSC. **(C)** Nomogram to predict the 1-, 3-, and 5-year overall survival rate of HNSC patients. **(D)** Calibration curve for the overall survival nomogram model in the discovery group. A dashed diagonal line represents the ideal nomogram. NCCGs, nerve-cancer cross-talk genes; HNSC, head and neck squamous cell carcinoma.

We used the clinicopathological features and the expression of seven genes in the model to establish a nomogram to predict the 1-, 3-, and 5-year survival rates. Univariate and multivariate analyses revealed the following independent prognostic factors: CHRNA5, L1CAM, CHRND, GRIN3A, age, M stage, and N stage (**Figures 5A, B** **)**. The nomogram is shown in **Figure 5C**, with a C index of 0.653. The nomogram could predict the 1-, 3-, and 5-year survival rates, which was close to the ideal model (**Figure 5D**).

### Relationship Between NCCGs and Immune Infiltration

**Figure 6:**
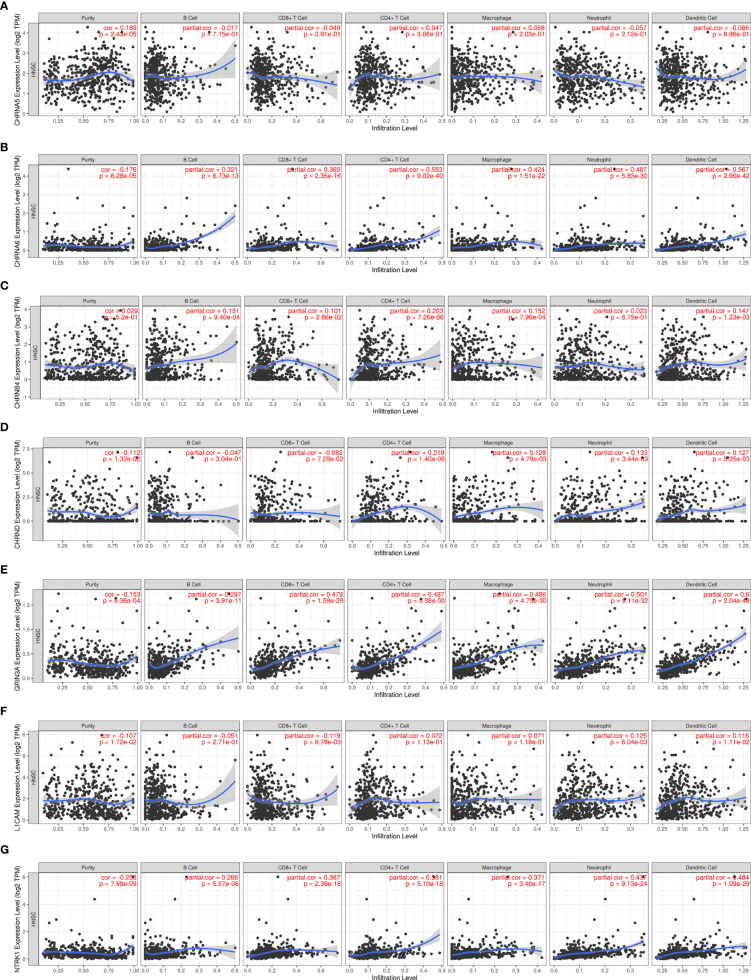
The association between seven prognostic NCCGs and immune infiltration. The association between the abundance of immune cells and the expression of CHRNA5 **(A)**, CHRNA6 **(B)**, CHRNB4 **(C)**, CHRND **(D)**, GRIN3A **(E)**, L1CAM **(F)**, and NTRK1 **(G)** in HNSC. NCCGs, nerve-cancer cross-talk genes; HNSC, head and neck squamous cell carcinoma.

Nerve is an important part of the tumor microenvironment, and there is a close relationship between the nerve system and the immune system. In our study, we used the TIMER to explore the association between seven genes in the prognostic gene model and immune infiltration, shown in **Figures 6A–G** and **Table 1**.

**Table 1:** The association between seven prognostic NCCGs and immune infiltration.

Gene	Purity	B cell	CD8+T cell	CD4+T cell	Macrophage	Neutrophil	Dendritic cell
CHRNA5	0.189^***^	−0.017	−0.049	0.047	0.058	−0.057	−0.006
CHRNA6	−0.176^***^	0.321^***^	0.365^***^	0.553^***^	0.424^***^	0.487^***^	0.567^***^
CHRNB4	0.029	0.151^***^	0.101^*^	0.203^***^	0.152^***^	0.023	0.147^**^
CHRND	−0.112^*^	−0.047	−0.082	0.218^***^	0.128^**^	0.133^**^	0.127^**^
GRIN3A	−0.153^***^	0.297^***^	0.479^***^	0.487^***^	0.486^***^	0.501^***^	0.6^***^
L1CAM	−0.107^*^	−0.051	−0.119^**^	0.072	0.071	0.125^**^	0.116^*^
NTRK1	−0.256^***^	0.206^***^	0.387^***^	0.381^***^	0.371^***^	0.437^***^	0.484^***^

^*^p < 0.05; ^**^p < 0.01; ^***^p < 0.001.

**Figure 6A** shows a positive correlation between CHRNA5 and tumor purity (cor = 0.189). **Figure 6B** shows that CHRNA6 was positively correlated with B cells (cor = 0.321), CD8+T (cor = 0.365), CD4+T (cor = 0.553), macrophages (cor = 0.424), neutrophils (cor = 0.487), and dendritic cells (cor = 0.567) and negatively correlated with purity (cor = −0.176). **Figure 6C** shows that CHRNB4 was positively correlated with B cells (cor = 0.151), CD8+T (cor = 0.101), CD4+T (cor = 0.203), macrophages (cor = 0.152), and dendritic cells (cor = 0.147). **Figure 6D** shows that CHRND was positively correlated with CD4+T (cor = 0.218), macrophages (cor = 0.128), neutrophils (cor = 0.133), and dendritic cells (cor = 0.127) and negatively correlated with purity (cor = −0.112). **Figure 6E** shows that GRIN3A was positively correlated with B cells (cor = 0.297), CD8+T (cor = 0.479), CD4+T (cor = 0.487), macrophages (cor = 0.486), neutrophils (cor = 0.501), and dendritic cells (cor = 0.6) and negatively correlated with purity (cor = −0.153). **Figure 6F** shows that L1CAM was positively correlated with neutrophils (cor = 0.125) and dendritic cells (cor = 0.116) and negatively correlated with CD8+T (cor = −0.119), purity (cor = −0.107). **Figure 6G** shows that NTRK1 was positively correlated with B cells (cor = 0.206), CD8+T (cor = 0.387), CD4+T (cor = 0.381), macrophages (cor = 0.371), neutrophils (cor = 0.437), and dendritic cells (cor = 0.484) and negatively correlated with purity (cor = −0.256). In conclusion, our results showed that there is a close relationship between NCCGs and immune infiltration.

### The Relationship Between NCCGs and TMB, MSI, and Drug Sensitivity

**Figure 7:**
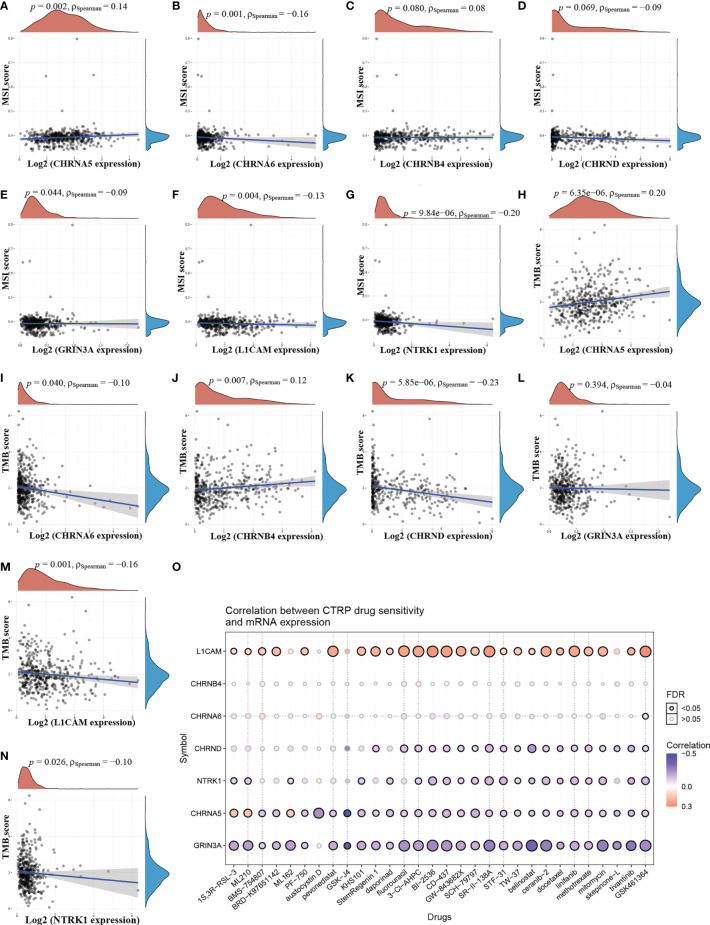
The relationship between NCCGs and TMB, MSI, and drug sensitivity. The correlation between MSI and CHRNA5 **(A)**, CHRNA6 **(B)**, CHRNB4 **(C)**, CHRND **(D)**, GRIN3A **(E)**, L1CAM **(F)**, and NTRK1 **(G)** in HNSC. The correlation between TMB and CHRNA5 **(H)**, CHRNA6 **(I)**, CHRNB4 **(J)**, CHRND **(K)**, GRIN3A **(L)**, L1CAM **(M)**, and NTRK1 **(N)** in HNSC. **(O)** The correlation between seven prognostic NCCGs and drug sensitivity in CTRP database. TMB, tumor mutation burden; MSI, microsatellite instability; CTRP, cancer therapeutics response portal; NCCGs, nerve-cancer cross-talk genes; HNSC, head and neck squamous cell carcinoma.

TMB and MSI are predictive biomarkers of immunotherapy (20). As shown in **Figure 7A**, there was a positive correlation between CHRNA5 and MSI. **Figures 7B, E–G** showed a negative correlation between CHRNA6, GRIN3A, L1CAM, NTRK1, and MSI. There were no significant relationship between CHRNB4, CHRND and MSI (**Figures 7C, D**). As shown in **Figures 7H, J**, there were positive correlations between CHRNA5, CHRNB4, and TMB (**Figure 7I, K, M, N**) showed a negative correlation between CHRNA6, CHRND, L1CAM, NTRK1, and TMB. But there is no significant relationship between GRIN3A and TMB (**Figure 7L**).

To further explore the potential of the above genes as therapeutic targets, we explored the relationship between gene expression and drug sensitivity in pan-cancer. Our data showed that drug sensitivity was positively correlated with LICAM and negatively correlated with CHRND, NTRK1, CHRNA5, and GRIN3A (**Figure 7O**). There was no significant relationship between CHRNA6, CHRNB4, and drug sensitivity.

### The Relationship Between NCCGs and HNSC Clinical Stage

**Figure 8:**
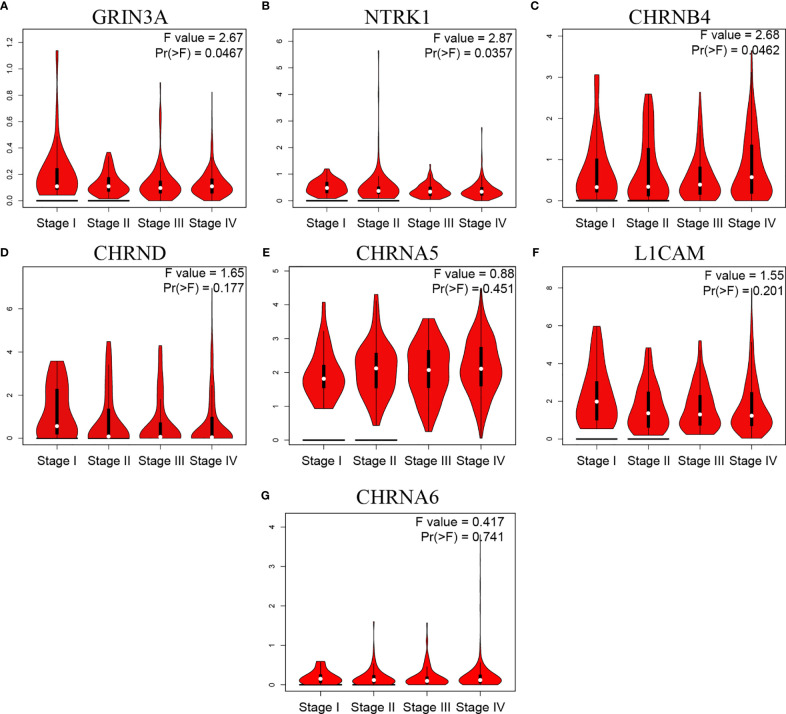
The relationship between seven prognostic NCCGs and HNSC clinical stage. Expression of GRIN3A **(A)**, NTRK1 **(B)**, CHRNB4 **(C)**, CHRND **(D)**, CHRNA5 **(E)**, L1CAM **(F)**, and CHRNA6 **(G)** in different stages in HNSC. NCCGs, nerve-cancer cross-talk genes; HNSC, head and neck squamous cell carcinoma.

Gene expression in tumor is closely related to clinical progress. We then analyzed the relationship between NCCGs and the clinical stage. The data showed that GRIN3A, NTRK1, and CHRNB4 were associated with stage (**Figures 8A–C**). But we found no correlation between CHRND, CHRNA5, L1CAM,CHRNA6 and stage (**Figures 8D–G**).

### The Expression of NCCGs in the Tumor Microenvironment of HNSC

Our study has shown that seven prognostic genes in the model are highly expressed in HNSC compared with normal tissue. However, it is not clear which cells these genes play a role in. Therefore, we used single-cell sequencing data to explore the expression of genes in different cells in the HNSC microenvironment.

**Figure 9:**
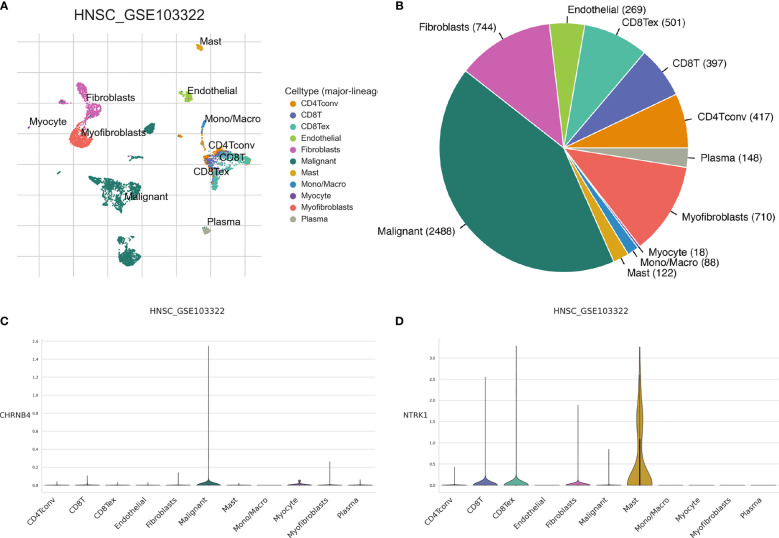
The expression of seven prognostic NCCGs in the tumor microenvironment of HNSC. **(A, B)** Cell types in HNSC single-cell sequencing data set GSE103322. **(C)** Average expression of CHRNB4 in different kinds of cells. **(D)** Average expression of NTRK1 in different kinds of cells. NCCGs, nerve-cancer cross-talk genes; HNSC, head and neck squamous cell carcinoma.

As shown in **Figures 9A, B**, there are myofibroblasts, malignant, plasma, fibroblasts, myocyte, mono/macro, endothelial, mast cell, CD8T, CD8Tex, and CD4Tconv in the microenvironment of HNSC. For CHRNB4, it is mainly expressed in the malignant cells (**Figure 9C**). NTRK1 gene is highly expressed in mast cell and slightly expressed in CD8+T and fibroblasts (**Figure 9D**). CHRNA5, CHRNA6, CHRND, L1CAM, and GRIN3A are commonly expressed, as shown in **Supplementary Figure S1**.

Therefore, the following focus on exploring the function of CHRNB4 in malignant cell and NTRK1 in mast cell.

### To Identify the Function of CHRNB4 in Malignant Cell

Single-cell gene set enrichment analysis was used to explore the possible function of gene. The malignant cells with high expression and low expression of CHRNB4 were selected for gene set enrichment analysis of the KEGG pathway.

**Figure 10:**
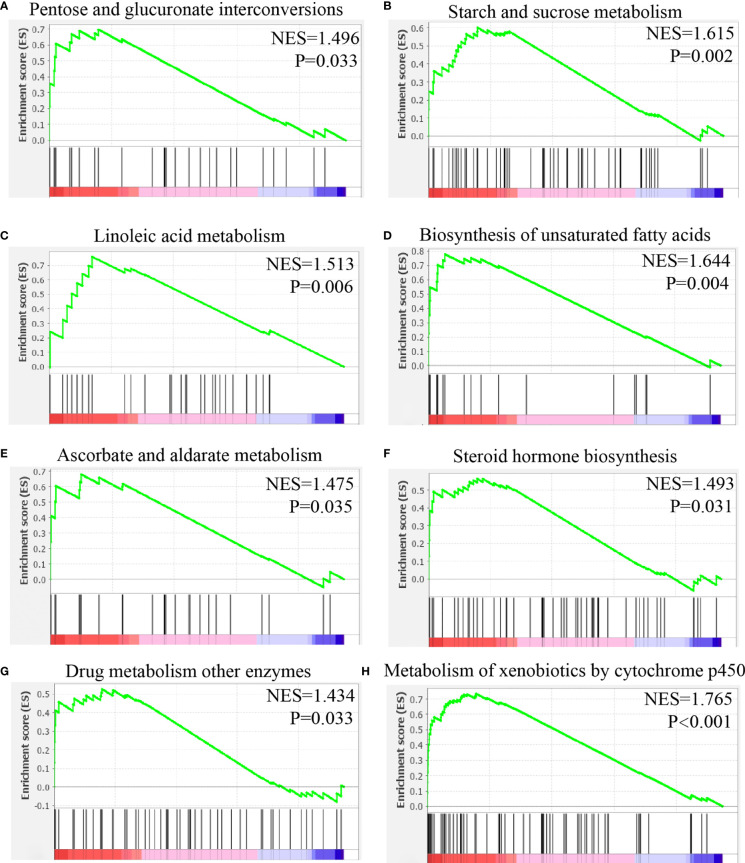
Gene set enrichment analysis of CHRNB4 in malignant cells of HNSC. **(A)** The expression of CHRNB4 positively correlated with the pentose and glucuronate interconversions. **(B)** The expression of CHRNB4 positively correlated with the starch and sucrose metabolism. **(C)** The expression of CHRNB4 positively correlated with the linoleic acid metabolism. **(D)** The expression of CHRNB4 positively correlated with the biosynthesis of unsaturated fatty acids. **(E)** The expression of CHRNB4 positively correlated with the ascorbate and aldarate metabolism. **(F)** The expression of CHRNB4 positively correlated with the steroid hormone biosynthesis. **(G)** The expression of CHRNB4 positively correlated with the drug metabolism other enzymes. **(H)** The expression of CHRNB4 positively correlated with the metabolism of xenobiotics by cytochrome p450.

The results showed that the following pathways were activated in malignant cell with high expression of CHRNB4: pentose-glucose conversion, starch-sucrose metabolism, linoleic acid metabolism, unsaturated fatty acid biosynthesis, ascorbic acid and lactic acid metabolism, steroid hormone biosynthesis, drug metabolism, and P450 metabolism of external substances (**Figure 10**).

### To Identify the Function of NTRK1 in Mast Cell

Mast cells with high and low expressions of NTRK1 were selected for gene set enrichment analysis of the KEGG pathway.

**Figure 11:**
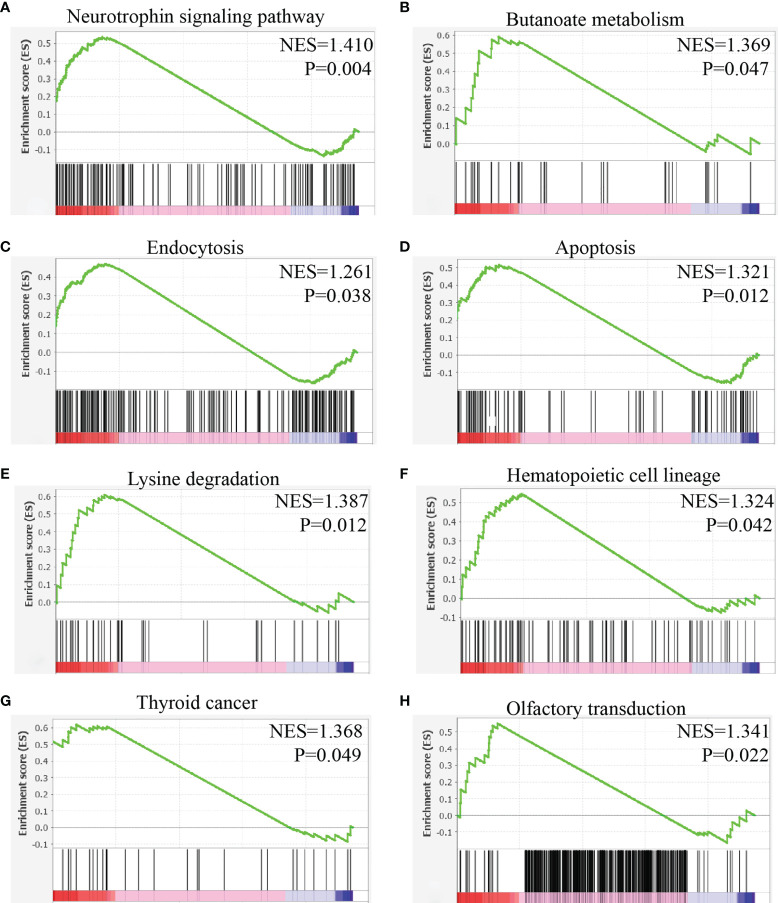
Gene set enrichment analysis of NTRK1 in mast cells of HNSC. **(A)** The expression of NTRK1 positively correlated with the neurotrophin signaling pathway. **(B)** The expression of NTRK1 positively correlated with the butanoate metabolism. **(C)** The expression of NTRK1 positively correlated with the endocytosis. **(D)** The expression of NTRK1 positively correlated with the apoptosis. **(E)** The expression of NTRK1 positively correlated with the lysine degradation. **(F)** The expression of NTRK1 positively correlated with the hematopoietic cell lineage. **(G)** The expression of NTRK1 positively correlated with the thyroid cancer. **(H)** The expression of NTRK1 positively correlated with the olfactory transduction.

The results showed that mast cells with high expression of NTRK1 were activated in the following pathways: neural tyrosine signal pathway, butyric acid metabolism, endocytosis, apoptosis, lysine degradation, hematopoietic system lineage, thyroid cancer, and olfactory conduction (**Figure 11**).

## Discussion

Nerve-cancer cross-talk plays an important role in tumorigenesis and development: neurotransmitters such as catecholamine and acetylcholine released by nerve activate the membrane receptors of cancer cells, stromal cells, and immune cells. On the other hand, neurotrophic factors secreted by cancer cells recruit axon and promote the growth of nerve (21). The research of Magnon et al. confirmed the important role of nerve in tumorigenesis for the first time (22). Pundavela et al. confirmed that cancer cells can promote the growth of nerve axon by releasing nerve growth factor precursor (pro-NGF) (23).

We defined 42 NCCGs through literature review, but the role of NCCGs in HNSC has not been elucidated. We obtained the genes with prognostic value through differential expression and K-M plotter and constructed an effective prognostic gene model based on these genes. We then found the relationship between key genes and immune infiltration, TMB, MSI, and drug sensitivity. Finally, through the single-cell sequencing data of HNSC, we showed the expression of key genes in different kinds of cells and explored the possible pathways of key genes in the high-expressed cells.

We first demonstrated the expression, mutation, and prognostic value of NCCGs. There were 23 differentially expressed genes in NCCGs, of which 10 genes were associated with prognosis. In HNSC, upregulation of CHRNA1, CHRNA5, CHRNB4, CHRND, CHRNG, and LICAM and downregulation of CHRNA6, GFRA2, GRIN3A, and NTRK1 indicate poor prognosis. The high expression of CHRNA1 is related to the low postoperative survival probability in early lung adenocarcinoma (24). Also, knockdown of CHRNA1 can reduce the drug resistance of EGFR mutant cell line PC9 to EGFR-TKI (25). The rs16969968 polymorphism of CHRNA5 is a risk factor for HNSC (26). Li et al. proved that CHRNB4 knockdown can inhibit the proliferation of esophageal squamous cell carcinoma *via* the Akt/mTOR and ERK1/2/mTOR pathways by cell counting kit-8, cloning formation assay, and Western blot (27). The overexpression of CHRNG indicates the possibility of sarcoma in children (28). Epidemiological studies have shown that CHRNA6 mutations can increase the susceptibility to esophageal squamous cell carcinoma (29). NTRK1 gene rearrangement can promote tumor progress and drug resistance in lung cancer (30).

We also discussed the gene expression in nonmalignant lesions and normal tissues, taking OLP as an example. Oral lichen planus is a precancerous lesion of oral squamous cell carcinoma (OSCC), which is considered to be a chronic inflammatory response mediated by T cells (31). We combined two OLP data sets (GSE52130 and GSE38616) including 14 disease samples and 14 healthy tissues and found 665 differential genes, of which only NTRK1 was in NCCGs (**Supplementary Table S5**). This suggested that most of the NCCGs differentially between HNSC and normal tissues are tumor-specific.

We then analyzed the GO function enrichment and KEGG signal pathway enrichment of NCCGs. NCCGs are mainly concentrated in membrane potential regulation, ion channel complex, axon formation, neuroreceptor activation, neuroactive receptor-ligand interaction, calcium signal pathway, and so on. Recent studies have shown that change in cell membrane potential can affect the growth of cancer cell (32). Calcium signal is associated with uncontrolled proliferation and invasiveness of cancer cell (33).

LASSO regression was used to construct a prognostic gene model based on seven genes (NTRK1, L1CAM, GRIN3A, CHRNA5, CHRNA6, CHRNB4, CHRND). This model can divide patients into high-risk group and low-risk group. In the external data verification, the same results were obtained as the training data. Nomogram can also be used to predict the 1-, 3-, and 5-year survival rates of patients. Liu et al. (34), Yao et al. (35), and Wang et al. (36) constructed a model based on genes to predict OS in HNSC, and AUC of models were 0.642, 0.709, and 0.8298, respectively. Compared with these studies, our AUC 0.634 of prediction for the 5-year OS is relatively poor. However, it is generally believed that the model has a certain distinguishing ability when the AUC is in the range of 0.6–0.75 (37). Published prognostic models were based on different types of gene sets, such as immunity, metabolism, pyroptosis, and autophagy. According to us, we are the first model based on a limited number of nerve-cancer cross-talk-related genes. In particular, combined with single-cell sequencing data, we analyzed the expression of prognostic markers in different cell groups in the HNSC microenvironment and located the therapeutic targets to specific cell groups. Our study suggests the potential of CHRNB4 as a target for direct regulation of HNSC tumor cells and the potential of NTRK1 as a target for regulation of mast cells.

Neurotransmitters released by nerve can affect a variety of immune cells. In the breast cancer model, the adrenal signal can increase the number of tumor-associated macrophages (38). In our study, we found the correlation between NCCGs and immune infiltration, which provides evidence for the nerve system regulating the immune microenvironment.

Using the data of transcriptome, we found that the expression of CHRNB4 and NTRK1 gene was significantly correlated with the clinical stage. However, we neither know in which type of cell these genes are mainly expressed nor the function of these genes. Based on the single-cell sequencing data of HNSC, we found that CHRNB4 was mainly expressed in cancer cells, while NTRK1 was mainly expressed in mast cells. This suggests that CHRNB4 may promote tumor development by directly affecting tumor cells, and NTRK1 may change the tumor microenvironment by affecting mast cells.

CHRNB4 is the coding gene of the N-type acetylcholine receptor β4 subunit (39). N-type acetylcholine receptor is a ligand-gated cationic channel (Na^+^, K^+^), which is divided into homopentamer and heteropentamer (40). The increase of the expression of the β4 subunit can increase the proportion of β4 in the heteropentamer, and then improve the sensitivity of the receptor to acetylcholine (41). Our results showed that CHRNB4 was highly expressed in HNSC, which may increase sensitivity of HNSC to acetylcholine released by nerve. Then acetylcholine changes the concentration of intracellular Na^+^ and K^+^ *via* N-type acetylcholine receptor. Interestingly, studies in T cells have shown that concentration of intracellular K^+^ can affect metabolism and then affect function of T cells (42, 43). We analyzed HNSC cells with different levels of CHRNB4 expression and found those differential genes were enriched in pentose-glucose conversion, starch-sucrose metabolism, linoleic acid metabolism, unsaturated fatty acid biosynthesis, ascorbic acid and lactic acid metabolism, steroid hormone biosynthesis, and so on. Taken together, CHRNB4 may affect the metabolic pathway. The possible mechanism is changing the concentration of intracellular K^+^ by increasing the sensitivity to acetylcholine.

NTRK1 encodes TRKA protein, which can be activated by nerve growth factor (NGF), thus affecting MAPK, PI3K, and PKC pathways (44). Compared with mast cells with low expression of NTRK1, pathways activated in mast cells with high expression of NTRK1 are as follows: neuronal tyrosine signal, butyric acid metabolism, endocytosis, apoptosis, and lysine degradation. Our results suggested that these pathways may be the mechanism of NTRK1 affecting mast cells.

The limitation of our study is that only transcriptome sequencing and single-cell sequencing data were used for analysis, lacking experimental support. In the future, we will use *in vivo* and *in vitro* experiments to further verify the role of NCCGs in HNSC.

In a word, we made a comprehensive bioinformatics analysis of NCCGs and constructed a seven-gene prognostic model (NTRK1, L1CAM, GRIN3A, CHRNA5, CHRNA6, CHRNB4, CHRND). Our findings provide insight into the molecular mechanism of the occurrence and development of HNSC and the identification of prognostic biomarkers and therapeutic targets.

## Data Availability Statement

The datasets presented in this study can be found in online repositories. The names of the repository/repositories and accession number(s) can be found in the article/**Supplementary Material**.

## Author Contributions

Conceptualization: JL and YX. Collection and assembly of data: GP. Data analysis and interpretation: JL. Software: YX. Writing—original draft preparation: JL and YX. Writing—review and editing: GW and LS. Visualization: KZ. Supervision: ZW. Project administration: GW and LS. Funding acquisition: GW and LS. All authors contributed to the article and approved the submitted version.

## Funding

This work was supported by the National Natural Science Foundation of China (No. 82002617 to LS and No. 82073351 to GW).

## Conflict of Interest

The authors declare that the research was conducted in the absence of any commercial or financial relationships that could be construed as a potential conflict of interest.

## Publisher’s Note

All claims expressed in this article are solely those of the authors and do not necessarily represent those of their affiliated organizations, or those of the publisher, the editors and the reviewers. Any product that may be evaluated in this article, or claim that may be made by its manufacturer, is not guaranteed or endorsed by the publisher.
