# Supplementary figures and images for: Identification of the Nerve-Cancer Cross-Talk-Related Prognostic Gene Model in Head and Neck Squamous Cell Carcinoma
Source: Front Oncol. 2021 Nov 29;11:788671. doi: 10.3389/fonc.2021.788671 (PMC8666427; doi:10.3389/fonc.2021.788671)

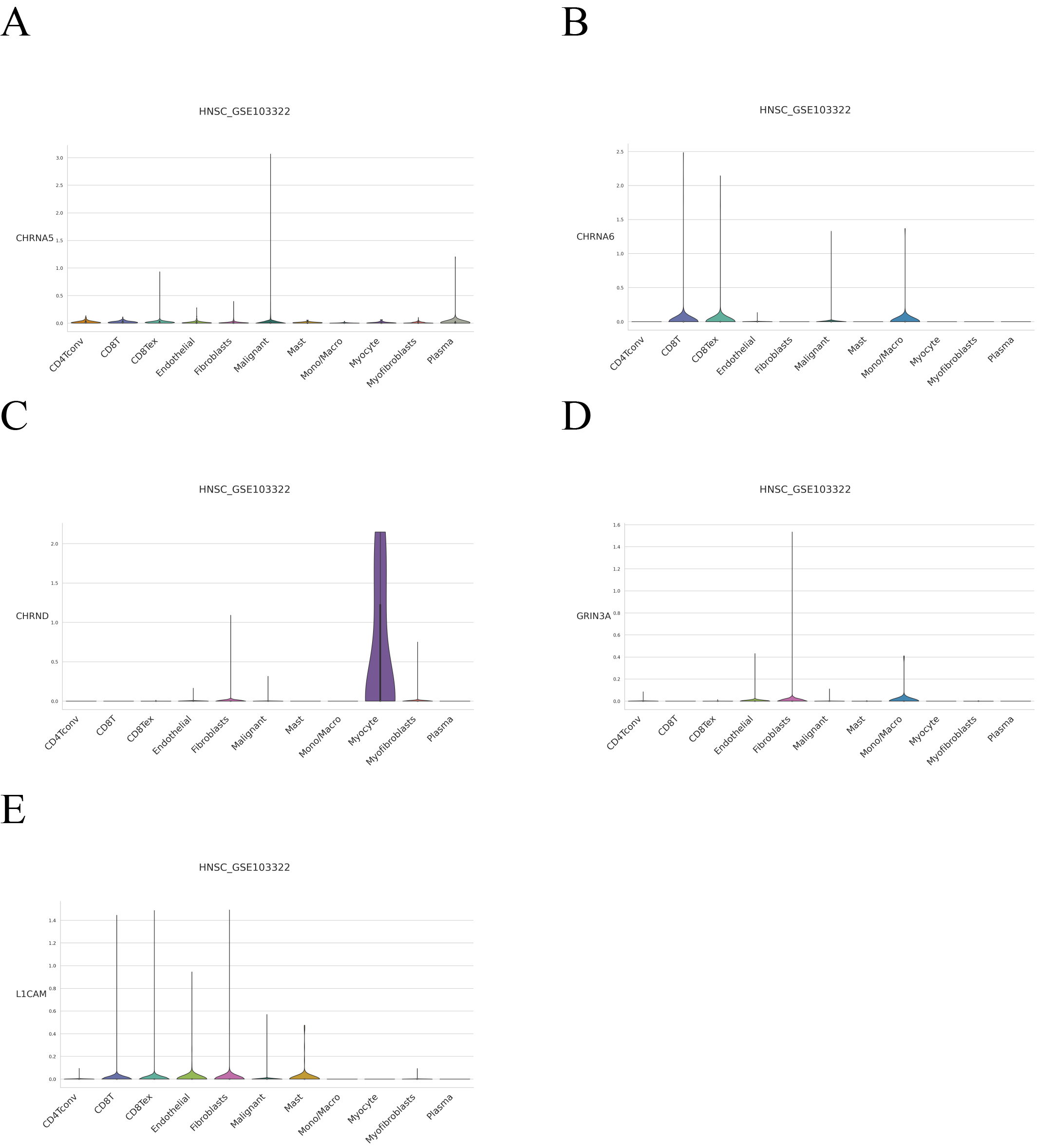

Supplement: Supplementary file 6 [file Image_1.tif]
